# Supplementary material for: The impact of early anti-SARS-CoV-2 antibody production on the length of hospitalization stay among COVID-19 patients
Source: Microbiol Spectr. 2023 Oct 9;11(6):e00959-23. doi: 10.1128/spectrum.00959-23 (PMC10715214; doi:10.1128/spectrum.00959-23)
Supplement: Supplemental file 1 — Table S1. [file spectrum.00959-23-s0001.docx]

Supplemental Table 1: Sociodemographic and Clinical characteristics of study participants, stratified by COVID-19 and PLWH/COVID-19.

| Characteristics | |  | Overall ^(1)^ | COVID-19 | PLWH/  COVID-19* | *P*  value ^(2)^ |
| --- | --- | --- | --- | --- | --- | --- |
|  |  |  |  |  |  |  |
| Gender | Male |  | 47 (55.3%) | 47 (55.3%) | 9 (60%) | 0.955 |
|  | Female |  | 38 (44.7%) | 38 (44.7%) | 6 (40%) |  |
| Skin Color | Brown |  | 57 (67.1%) | 57 (67.1%) | 14 (93.3%) | 0.207 |
|  | White |  | 13 (15.3%) | 13 (15.3%) | 1 (6.7%) |  |
|  | Black |  | 8 (9.4%) | 8 (9.4%) | 0 (0%) |  |
|  | Unk |  | 7 (8.2%) | 7 (8.2%) | 0 (0%) |  |
| State of Origin | RJ |  | 83 (97.6%) | 83 (97.6%) | 15 (100%) | 1 |
|  | AM |  | 2 (2.4%) | 2 (2.4%) | 0 (0%) |  |
| Schooling | High school |  | 32 (37.6%) | 32 (37.6%) | 7 (46.7%) | 0.251 |
|  | Elementary School |  | 18 (21.2%) | 18 (21.2%) | 2 (13.3%) |  |
|  | University Education |  | 8 (9.4%) | 8 (9.4%) | 0 (0%) |  |
|  | First grade |  | 16 (18.8%) | 16 (18.8%) | 6 (40%) |  |
|  | Illiterate |  | 6 (7.1%) | 6 (7.1%) | 0 (0%) |  |
| Age |  |  | 57 (IQR=23.51) | 60 (IQR=22.83) | 38 (IQR=18.22) | < 0.001 |
| Clinical data during COVID-19 | |  |  |  |  |  |
| Systemic arterial hypertension | No |  | 51 (51%) | 39 (45.9%) | 12 (80%) | 0.031 |
|  | Yes |  | 49 (49%) | 46 (54.1%) | 3 (20%) |  |
| Diabetes mellitus | No |  | 67 (67%) | 54 (63.5%) | 13 (86.7%) | 0.145 |
|  | Yes |  | 33 (33%) | 31 (36.5%) | 2 (13.3%) |  |
| COPD* | No |  | 90 (90%) | 76 (89.4%) | 14 (93.3%) | 1 |
|  | Yes |  | 10 (10%) | 9 (10.6%) | 1 (6.7%) |  |
| Current Smoking | No |  | 93 (93%) | 81 (95.3%) | 12 (80%) | 0.111 |
|  | Yes |  | 7 (7%) | 4 (4.7%) | 3 (20%) |  |
| Active Tuberculosis | No |  | 96 (96%) | 85 (100%) | 11 (73.3%) | < 0.001 |
|  | Yes |  | 4 (4%) | 0 (0%) | 4 (26.7%) |  |
| Treated Tuberculosis | No |  | 99 (99%) | 85 (100%) | 14 (93.3%) | 0.325 |
|  | Yes |  | 1 (1%) | 0 (0%) | 1 (6.7%) |  |
| Hepatic Cirrhosis | No |  | 99 (99%) | 85 (100%) | 14 (93.3%) | 0.325 |
|  | Yes |  | 1 (1%) | 0 (0%) | 1 (6.7%) |  |
| Dyspnea | Yes |  | 77 (77%) | 67 (78.8%) | 10 (66.7%) | 0.485 |
|  | No |  | 23 (23%) | 18 (21.2%) | 5 (33.3%) |  |
| Outcome | Discharge |  | 66 (66%) | 55 (64.7%) | 11 (73.3%) | 0.723 |
|  | Death |  | 34 (34%) | 30 (35.3%) | 4 (26.7%) |  |
| 1-Three individuals with unknown information about the HIV serology result.  2- *P-values* were calculated either by nonparametric Mann-Whitney (Wilcoxon rank-sum) test or Fisher’s exact test for nominal and continuous numeric variables, respectively.  * Note: PLWH/COVID-19 refers to people living with HIV and COVID-19, COPD= Chronic Obstructive Pulmonary Disease. Values are presented as median (interquartile range (IQR)), or number (percentage) | | | | | | |
