# Supplementary figures and images for: The impact of early anti-SARS-CoV-2 antibody production on the length of hospitalization stay among COVID-19 patients
Source: Microbiol Spectr. 2023 Oct 9;11(6):e00959-23. doi: 10.1128/spectrum.00959-23 (PMC10715214; doi:10.1128/spectrum.00959-23)

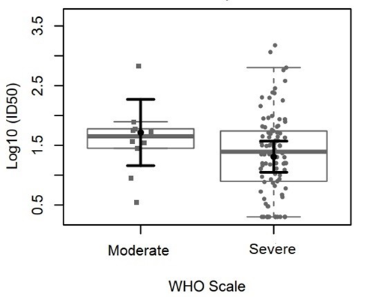

Supplement: Supplemental file 2 — Fig. S1. [file spectrum.00959-23-s0002.tif]
