# Supplementary material for: The impact of early anti-SARS-CoV-2 antibody production on the length of hospitalization stay among COVID-19 patients
Source: Microbiol Spectr. 2023 Oct 9;11(6):e00959-23. doi: 10.1128/spectrum.00959-23 (PMC10715214; doi:10.1128/spectrum.00959-23)
Supplement: Supplemental file 3 — Legend to Fig. S1. [file spectrum.00959-23-s0003.docx]

**Supplemental Figure 1**: PsV SARS-CoV-2 neutralizing antibody ID50 reciprocal plasma dilutions grouped by WHO classification as moderate and severe disease. The sample distributions of data are represented in box plots and strip plots in gray. In black, the center circle represents the expected mean marginal effect for each group estimated from linear multiple fixed effects models. The fixed effects were adjusted by age, gender, self-declared skin color, number of comorbidities, and days since the first symptoms of COVID-19 at hospital admission. Black horizontal bars represent the 95% confidence intervals of the mean expected marginal effects by group.
